# Famous English Physicians

**Published:** 1910-05

**Authors:** 


A Monthly Review of Medicine and Surgery.

*EDITOR*

WILLIAM WARREN POTTER, M. D.

All communications, whether of a literary or business nature, books for review and xchanges, should be addressed to the editor, 238 Delaware Avenue, Buffalo, N.Y.

Vol. Lxv. MAY, 1910. No. 10

### Famous English Physicians

Mr. George W. Smalley, in contributing the series of Anglo-American Memories to *The Tribune,* recently discoursed interestingly of three famous English physicians. We feel sure our readers will enjoy these anecdotes, and that Mr. Smalley will pardon us for reproducing them. They deserve preservation in medical literature and to be incorporated in the biographies of these celebrated medical men.

### SIR THOMAS BARLOW.

Coming to England in the summer of 1896 on a holiday, I had some slight illness and asked a friend whom I should consult. My own doctor was by that time attending patients, I suppose, in another and better world. My friend said he had lately seen fourteen physicians about his son, and each of the fourteen had given a different name to his son's disease.

Then I went to Dr. Barlow, who said, after a long examination, "I do not know what is the matter with your son nor what to prescribe for him." Then I felt I had found a doctor whom I could trust.

So I went to Dr. Barlow, without an introduction. At the end of a rather long consultation and a definite opinion and a settled prescription, I asked what his fee was.

"Nothing."

I thought he had misunderstood my question, and repeated it.

"Nothing. I can take no money from a man who has done as much as you have to keep the peace between the two countries."

When I next saw the manager of *The Times* I told him of this incident, which he seemed to think interesting. He said:

"Such evidences of good feeling from a man so distinguished as Barlow and so far removed from politics do indeed make for good feeling on both sides. I hope you will tell all your own people."

It is difficult, for I cannot tell it without more or less directly paying a compliment to myself. But many years have since ebbed away. Modesty is at best but an inconvenient handmaiden, from whom I would part company if I could. Let her keep to her proper place. An obligation of honor is peremptory; and this, perhaps, is one. I did tell a certain number of friends at the time. and now I repeat the anecdote to a larger number. I set it against Mr. Price Collier's mischievous dictum that English and Americans do not like each other. The dictum already seems to belong to a distant and misty and mythical past.

Since that year of 1896 Dr. Barlow has become (in 1902) Sir Thomas Barlow, Bart., and Physician to the King's Household—about as high as anybody can go in the medical profession. A Lancashire lad to begin with, he has had a vast hospital experience, and still keeps up his hospital work; he has a vast private practice; Harvard and two Canadian universities have give him their LL. D.; he is an F. R. S., a K. C. V. O., and other parts of the alphabet pay him tribute. All these and many other titles and distinctions have their value, though the late Sir Henry Drummond Wolff, who had more than most men, did say: "They give me every kind of letter to my name except L. S. D." But the essential thing in Sir Thomas Barlow's case is that he has the confidence of the public and of his profession.

Sir Thomas was one of the medical advisers of the King at the time of the operation for appendicitis by Sir Frederick Treves. Those were critical and fateful days, about which many legends have since grown up. The one I like best has nothing to do with Sir Thomas Barlow, nor have I ever asked him whether it is true. nor would he tell me if I did. But even a legend may be true, and this I believe is.

### SIR FREDERICK TREVES.

Sir Frederick Treves, in one of the quaint phrases of the older days, Serjeant-Surgeon to the King, and reckoned, perhaps, the most brilliant operator of his time, was summoned to the King on a Monday in June, 1902. He made his examination and told the King there must be an operation on the following day. The right technical name for the King's malady is not appendicitis, which has become a household word, but perityphlitis. The King assented in principle to the operation, but said to Sir Frederick Treves it could not be performed before Wednesday. Answered Sir Frederick very gravely:

"Sir, I shall not be here on Wednesday."

The audacity of the reply amazed the King and filled the bystanders with terror. The King asked:

"What do you mean?"

"Sir, with every respect, I mean that I shall not be here on Wednesday."

"Am I to understand that if I wish you to perform the operation on Wednesday you will not do it?"

"I am obliged to tell your majesty that, in my judgment, if the operation is to be successful it must be done on Tuesday. If not on Tuesday it must be by another hand than mine."

The King finally yielded; the operation took place on Tuesday; the success was complete. But here again gossip steps in, alleging that the first incision revealed such results as to startle some of the few professionals present. At least one of them protested against going on, and finally left the room, saying hotly he would not stay to see the King "cut to pieces." But nothing shook Sir Frederick's confidence or nerve. He went on boldly, tranquilly, to the successful end.

Why, then, all this difference between Tuesday and Wednesday? Because, the surgeons will tell you, the King's malady was already so far advanced that a day's delay meant the danger, or the certainty, of blood poisoning. With this knowledge he was bound to face the King's anger. It was the way in which he faced it that made the act so impressive, and carried the day. The King with all his suavity and unfailing tact and kindness, is a King. He has in him a thousand years of kingly traditions and inheritances. When he was Prince of Wales, and only the first of his mother's subjects, he had them. They have grown stronger with use since he came to the throne. He will listen to reason, but decides for himself, as a king should, or any other man should. If Sir Frederick had begun by explaining his reasons, the king might easily enough have brushed them aside and ended the discussion with a regal: "Wednesday, then." Nothing would have impressed the king like the respectful, "Sir, I shall not be here on Wednesday"; respectful, but also extremely rebellious. The essence of devoted loyalty was there, all the same.

One thing, it seems to me, the great surgeons and physicians I have known had in common. They were great men, first of all. They had great qualities outside of their profession. Two years ago last September, a time when the big men are mostly away, I wanted a surgeon and knew not where to find one.

### SIR HENRY MORRIS.

A chemist finally gave me a name—Mr. Henry Morris—and an address; name wholly unknown to me, though the address—Cavendish Square—implied at least professional prosperity. I had had a fall at the Playhouse, as Mr. Maude calls his little theatre, the night before, leaving a box by what I supposed to be steps, and in the absence of steps coming down on the floor, bruised, and I know not what else. My surgeon made his examination. What struck me was that he wasted never a word, nor a gesture. The touch of his hands, of his fingers, had a mathematical or instrumental precision. So had his questions. In five minutes or less he had covered the ground and delivered his opinion. Anything might have happened, but nothing had—bar the bruised muscles. "We'll attend to those for you." He asked if I was leaving town, and when I said I was sailing for New York on Saturday he remarked:

"If you were a workingman I should send you to the hospital and you would be left in bed till you were well. But if you choose to sail on the Lusitania you must bear the pain. Now, as you are here, you might as well let me overhaul you."

Then, as before, the same precision, the same delicacy of touch, the same rapidity, nothing hurried, nothing missed; his examination a work of art as well as of science. Then he began to talk of other things; and again, and even stronger, was the impression of being in contact with a master mind. Seldom have I spent a more stimulating hour. He was, I found later, Mr. Henry Morris, Consulting Surgeon to the Middlesex Hospital and President of the Royal College of Surgeons. In other words, Mr. Henry Morris, about whom I ought to have known, but did not, was, and is, in the very front rank of his profession. His eminence has since been recognised and rewarded by the King, and he is now Sir Henry Morris, Bart. I suppose even a republican may admit that, if titles are to be conferred, they are well conferred on men eminent in science.

